# Supplementary material for: Repeatedly unreliable, and unreliably repeatable: the trials-to-criterion fallacy
Source: Anim Cogn. 2026 Jul 13;29(1):59. doi: 10.1007/s10071-026-02082-0 (PMC13473314; doi:10.1007/s10071-026-02082-0)
Supplement: Supplementary file 1 [file 10071_2026_2082_MOESM3_ESM.pdf]

Supplementary material for

Repeatedly unreliable, and unreliably repeatable: the trials-to-criterion fallacy

Animal Cognition (2026)

R. J. Dugand

Appendix 1 | Latent traits and their noisy, indirect measurements

Presented with a complex task, the number of seconds taken to solve the task (continuous response), or the proportion of successful attempts at solving the task (binomial response), may provide estimates of intelligence. While these responses appear directly in the data, intelligence itself does not. Intelligence is treated as the unobserved cause of those responses and is, therefore, a **latent trait** (see (Borsboom 2008)).

Importantly, when we attempt to measure latent traits, the responses that appear in the data are typically noisy estimates of those latent traits. Consequently, even when an underlying latent trait is perfectly stable and perfectly heritable, inflated measurement error sets an upper limit to metrics like repeatability and heritability (Dohm 2002).

To illustrate, consider a sporting analogy. How *fast* does Australian cricketer Mitchell Starc bowl? How *good* is Mitchell Starc? Bowling speed can be **measured directly** using a radar gun, and 'speed' appears in the data. However, his quality – a latent trait – must be inferred from his performance, which, after dominating England in the 2025/26 Ashes series, is 433 wickets [passes] from 20,013 balls [Bernoulli trials]; a strike rate (SR) of 46.2 balls per wicket (ESPNcricinfo 2026).

Assuming Mitchell Starc's bowling statistics exactly reflect his true underlying latent quality, we can ask what impact Bernoulli sampling might have had on his career (R code):

```
set.seed(56)
Nwickets <- replicate(1000, sum(rbinom(20013, 1, 433/20013)))
range(Nwickets)
# 372-499
```

Despite a massive sample size ($N > 20{,}000$), simulated careers of identical length and identical latent quality produced anywhere from 372 (SR ~ 54; worse than Brett Lee) to 499 wickets (SR ~ 40; better than Dale Steyn). This illustrates that even when the latent trait is fixed, indirect measurements based on repeated Bernoulli sampling inevitably contain substantial within-individual variability. Indirectly measuring latent traits can be noisy. Estimates of intelligence may be particularly noisy when measured with TTC.

Appendix 2 | Within-individual variability across learning trajectories

In the **Main Text**, I focused on a single learning trajectory (**Fig. 1**). In **Appendix 2**, I extend this, presenting data for a range of different of learning trajectories.

These simulations are presented in two sections. First, *p* is fixed for all trials for a given task; there is no learning. Second, *p* increases as the number of trials increases (*i.e.*, learning), based on various sets of conditions. All simulations are based on the 10/12 task structure, which are common parameters in animal cognition research.

In **Fig. 2** of the **Main Text**, I present a distribution of N_{TTC} scores that result from a single learning trajectory. In **Fig. S1** (left) and **S2**, below, each x-axis value essentially represents a single learning trajectory. The within-individual variability associated with that learning trajectory is represented by the distribution of values on the y-axis.

Appendix 2.1 | Fixed p: stochasticity without learning

My first goal was to demonstrate the variability in N_{TTC} scores that can result when the probability of success, *p*, never changes across trials. These simulations provide an important demonstration of how the proportion of tasks completed increases as the number of trials increases, and thus demonstrate the fallacy of TTC.

To implement these simulations, I used the custom TTC function, where the vector (sequence) of probabilities, *p*, that define the learning trajectory was a single probability, repeated indefinitely. For each value of $p = 0.5$, $p = 0.55$ ... $p = 1$, I performed 10,000 simulations. I then calculated interquartile ranges and 95% quantiles to represent the distributions of the simulated data. I also quantified the proportion of tasks that were completed after each number of trials for each value of *p*.

When $p \sim 1$, the N_{TTC} score mean and variability were low. As *p* decreased, the mean and variability increased dramatically (**Fig. S1**, left). This relationship between the probability of success, *p*, the mean number of trials, and score variability is a well-known property of negative binomial sampling. Unless the probability of success was high ($p > 0.75$), there was a large distribution of scores for a given probability of success (*e.g.*, for $p = 0.75$, the 95% intervals were 10-52). N_{TTC} scores were also right skewed; for example, with $p = 0.7$, the median score was 21, the 2.5% quantile was 10, and the 97.5% quantile was 75. This distribution starkly **resembles empirical TTC data** (**Fig. S2**), where most scores are low, and some are very high. If all individuals in a study acquire a task quickly, but make errors, then $p \sim 0.7$ makes intuitive and biological sense. **Because this biologically realistic scenario, where everyone is intelligent but makes errors, can reproduce the structure of real TTC data (Fig. S2), it provides the clearest illustration of why single estimates per individual should never be considered useful in individual-based studies**, and why demonstrating the repeatability of performance is essential.

Fig. S1 (right) shows the accumulation of completed tasks for each value of *p*. When $p = 0.5$, this directly demonstrates the accumulation of false positives described by Harrington and Lambert (2025). After 25 trials, the 'risk' of a false positive is ~10%; it reaches 50% by ~126 trials, and 95% by the ~600th trial. Eventually, all individuals pass by random chance (100% false positives). **The fallacy underlying TTC is that there is a <5% probability of an individual reaching the task criterion by chance.** This is only true for one window of trials. Citing the false positive risk in relation to the per-window risk is nonsensical.

Notably, when individuals are only slightly more likely to pass than fail in each trial, the proportion of completed tasks accumulates very quickly. For example, at $p = 0.6$, the 25th and 50th percentiles were just 22 and 42, respectively, demonstrating that that low scores (interpreted as high intelligence) are very common even if individuals are only slightly more likely to pass than fail.

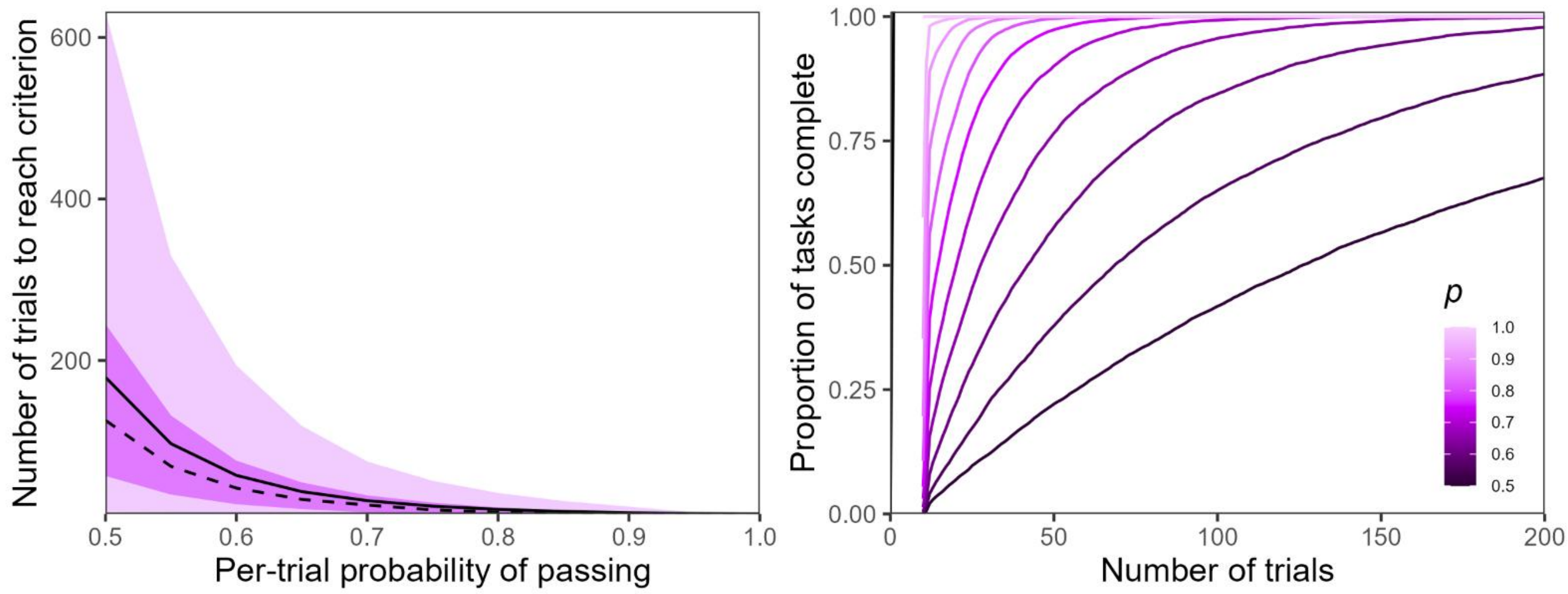

Fig. S1 | **Left**. Number of trials (N_{TTC}) to reach task criterion (N_{pass} = 10, N_{window} = 12) as a function of the probability of passing each trial, p. Dashed line is the median N_{TTC} score; solid line is the mean N_{TTC} score; dark band represents the interquartile range, and light band represents the 95% sampling intervals. **Right**. Proportion of 10,000 tasks completed after a given number of trials for different probabilities of success, p, where p = 0.5, 0.55...1.

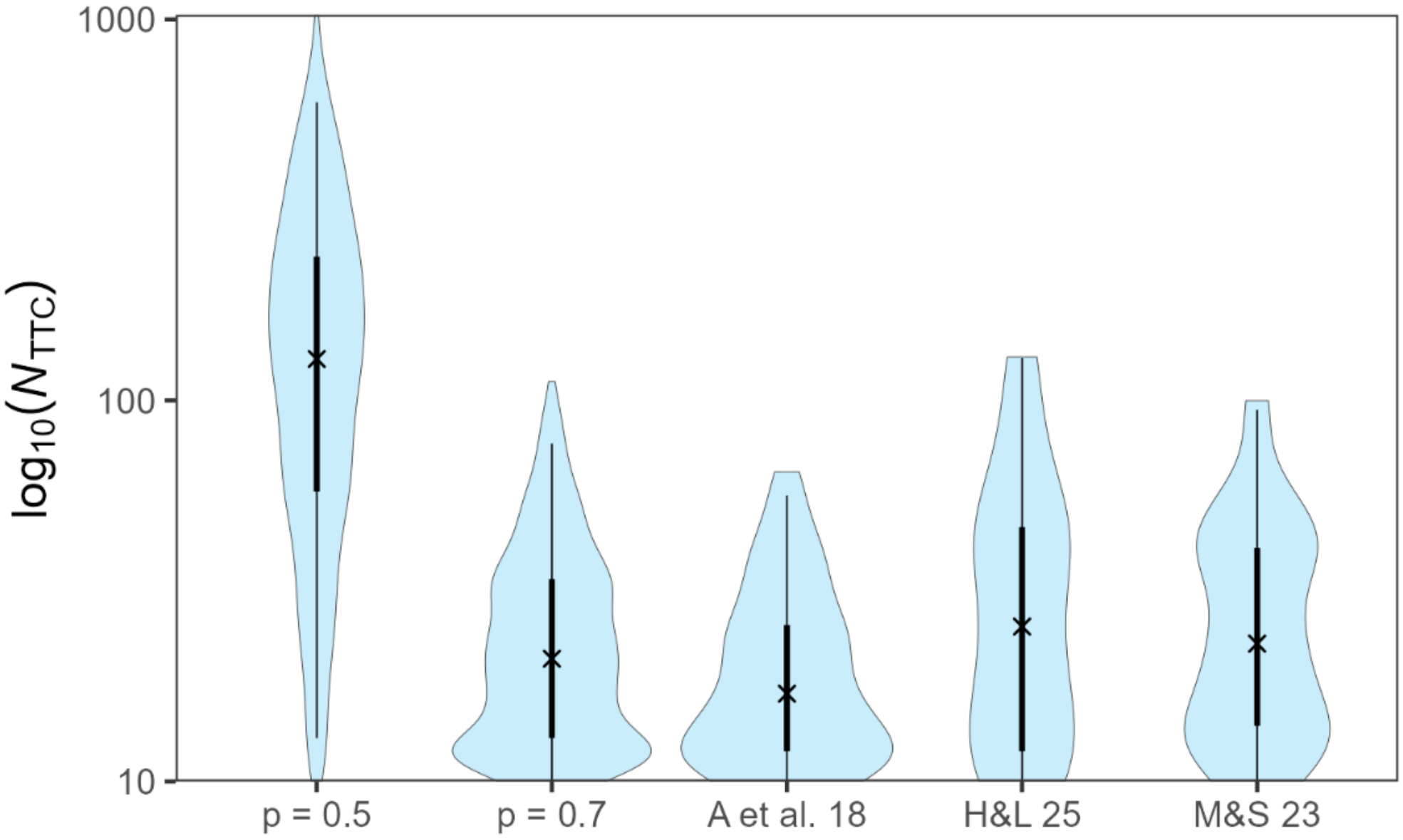

Fig. S2 | Distribution of N_{TTC} (number of trials to criterion) scores ($\log_{10}$) for simulated (p was fixed at either p = 0.5 or p = 0.7) and empirical (A et al. 18 = Ashton et al. 2018; H&L 25 = Harrington and Lambert 2025; M&S 23 = McCallum and Shaw 2023) TTC data. When p = 0.5, the distribution of N_{TTC} scores is considerably higher than empirical estimates. This categorically demonstrates that, for these empirical studies, individuals are performing better than chance, providing unequivocal evidence for learning at the population level. However, when p = 0.7, the simulated distribution of scores resembles empirical distributions **despite the absence of true between-individual differences**. Hence, although low mean scores is evidence for learning at the population level, between-individual differences may provide no insight about differences in cognitive abilities.

Appendix 2.2 | Variable p: stochasticity with learning

My next goal was to demonstrate the variability in N_{TTC} scores that can result when there is learning; that is, when p increases as a function of the number of trials.

Here, individuals start to learn the task after N_{guess} trials, after which p increases (from p_{min} = 0.5) towards a maximum value (p_{max}) until task acquisition (after N_{guess} + N_{learn} trials, where N_{learn} is the number of trials spent learning; see **Fig. 1** in the **Main Text**). I set this maximum value to be either p_{max} = 1 (perfect, no mistakes after task acquisition) or p_{max} = 0.8 (imperfect, 20% mistakes after task acquisition). Learning was either instant (N_{learn} = 0), fast (increasing at 0.02 per trial, such that N_{learn} = 15 for p_{max} = 0.8 or N_{learn} = 25 for p_{max} = 1), or slow (increasing at 0.01 per trial, such that N_{learn} = 30 for p_{max} = 0.8 or N_{learn} = 50 for p_{max} = 1).

In most research, individuals are allowed to interact with the task (*i.e.*, find the reward) before the task before the first trial. Hence, N_{guess} + N_{learn} = 0 is possible for 'highly intelligent' individuals; for these individuals, the minimum score is N_{pass} = 10. Thus, generally, I define deterministic reaching of the task criteria as: N_{guess} + N_{learn} + N_{pass} trials (see **Main Text** for full explanation). I use this definition to test the performance of the TTC method, representing N_{guess} + N_{learn} + N_{pass} by a red line in **Fig. S3**.

For each set of parameters, I simulated 10,000 performance scores, producing a total of 2 x 3 x 151 x 10,000 = 9,060,000 N_{TTC} scores.

As above, each x-axis value in **Fig. S3** essentially represents an 'individual', with variability in scores reflecting null variation that arises from stochasticity, and the red line is N_{guess} + N_{learn} + N_{pass}. Thus, TTC is accurate if the mean and median scores track the red line, and precise if there is limited scatter of values around the red line.

Across most parameter sets, the mean and median N_{TTC} scores were below the red line (inaccurate and downwardly biased, left skewed; **Fig. S3**). Only when there were few guesses (small values of N_{guess}) and learning was instant (N_{learn} = 0) and perfect (p_{max} = 1) were scores accurate (along the red line) and precise (low variability). Any deviation from the ideal learning parameters massively inflated within-individual variability (low precision). The ~2% probability of guessing 10 out of 12 correctly is evidenced by the lower 2.5% sampling interval being just above 12 across all parameters. Likewise, the accumulated false positive risk while p = 0.5 ensures that the 25th percentile was never higher than 57.

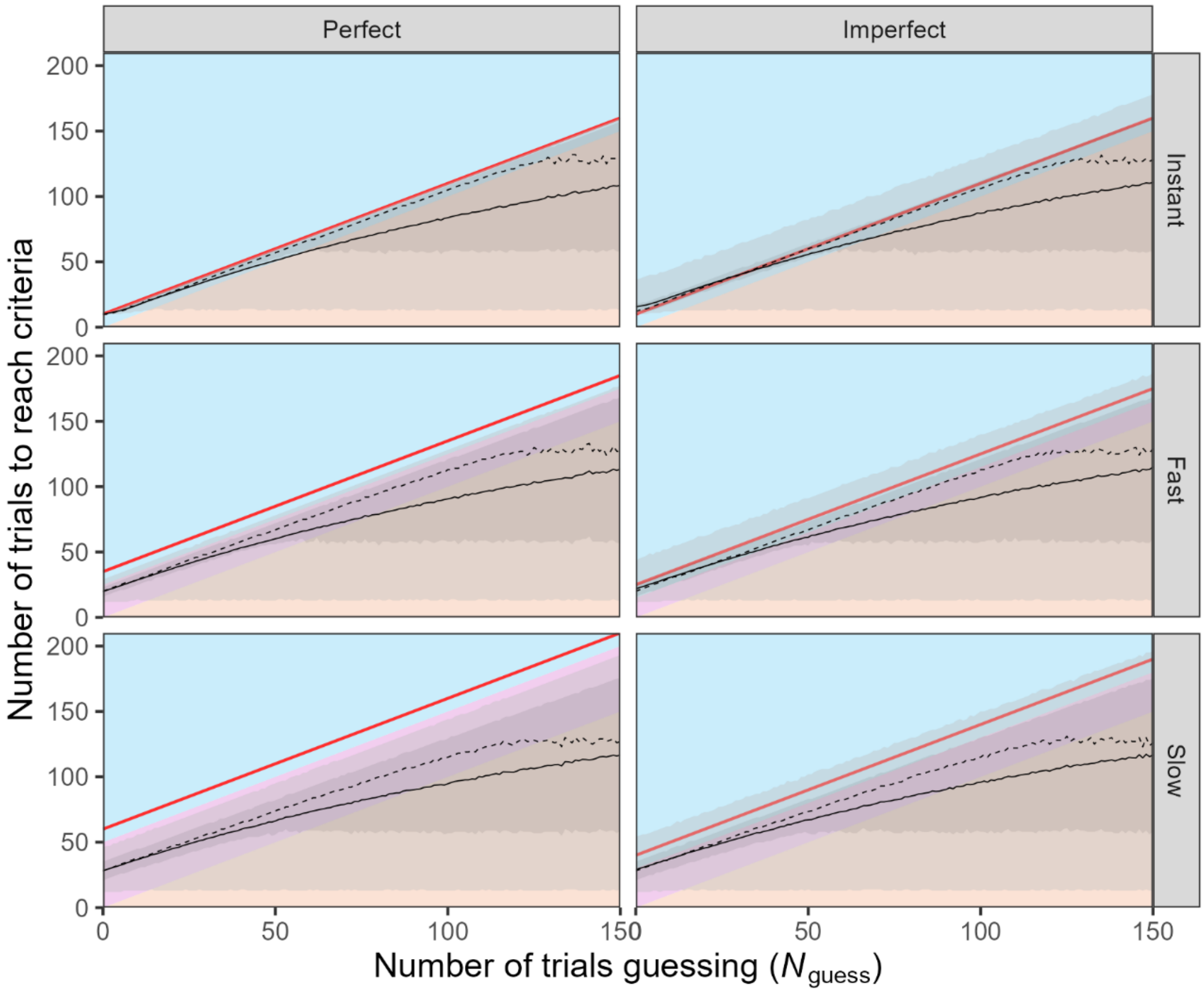

Fig. S3 | Number of trials to reach criteria as a function learning speed (instant, fast, or slow) and probability of passing in the mastery phase (perfect [p_{max} = 1] or imperfect [p_{max} = 0.8]) parameters. Across 10,000 simulations per parameter set and value of N_{guess} (number of guesses, where p_{min} = 0.5): black line is the mean score; dashed line is the median score; dark grey band is the interquartile range (25-75%); light grey band is the 95% sampling intervals (2.5-97.5%). Background colours represent the three phases (guessing = orange, learning = pink, mastery = light blue) in the associated trial number (see **Figure 1** in the **Main Text**). The red line represents N_{guess} + N_{learn} + N_{pass}, which represents deterministic reaching of the task criterion, and is what TTC should accurately and precisely estimate.

Appendix 3 | Repeatability of N_{TTC} scores: binomial houses with moving windows have low ceilings

Appendix 3.1 | Null distribution of repeatability with fixed p

To be confident that between-individual differences are real, not stochastic variability, it is necessary to demonstrate that N_{TTC} performance scores are repeatable. If many individuals are each measured multiple times (at least twice), then variability in scores can be partitioned into within- and between-individual differences. The proportion of variance that is attributable to between-individual differences is the repeatability.

In **Appendix 3.2** below, I simulate between-individual differences and calculate repeatability, similar to the **Main Text**. Here, however, my objective is to demonstrate the null distribution of repeatability that can result when all individuals have the **same fixed learning trajectory**. In other words, if all individuals were exactly the same, what is the null distribution of repeatability estimates? Generating a null distribution of repeatability allows us to determine how high repeatability needs to be to be confident that we are capturing true between-individual differences in performance scores.

To assess the null distribution of repeatability, I used the data generated from the single trajectory in the **Main Text** and, separately, the data generated for the fixed probability with $p = 0.7$. For each, I randomly sampled either two or four ($N_{replicates} = 2$ or 4) N_{TTC} scores for each of 10, 25, 50, 100, or 200 individuals ($N_{individuals} = 10, 25, 50, 100$, or 200). I then fit a linear model using *lme4* (Bates et al. 2015), with individual ID fit as a random effect and N_{TTC} score as the response variable. This partitions variation into within- and between-individual differences in scores, from which I calculated the repeatability (see **Main Text** for full details). I repeated the approach 1,000 times per parameter set (2 x 2 x 5 x 1,000 = 20,000 simulations in total), from which I calculated median repeatability and 95% sampling intervals.

The median repeatability was always ~0, demonstrating that more than half of all random data sets would not return spurious repeatability. However, 97.5% quantiles were all >0, were relatively high when individuals were only measured twice (**Fig. S4**), and were as high as ~0.6 when only 10 individuals were measured. This demonstrates that moderate repeatability can readily result despite the absence of true between-individual differences. Spurious repeatability was substantially reduced when $N_{individuals} > 25$ and $N_{replicates} = 4$, though the 97.5% sampling interval was almost always at least as high as ~0.1. Results were consistent whether there was learning (**Fig. 1** individual) or not (fixed, $p = 0.7$).

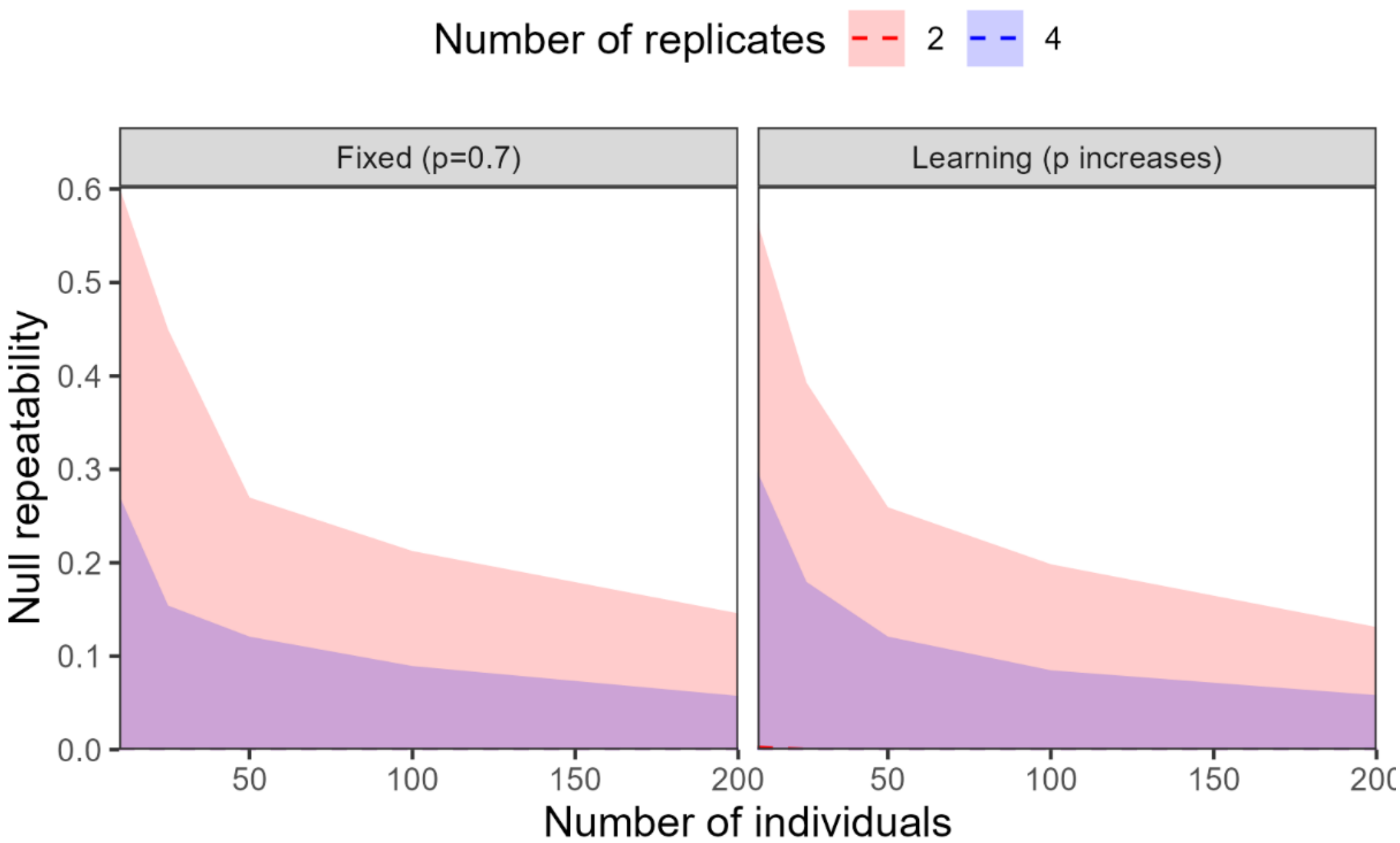

Fig. S4 | Null distributions for repeatability when all individuals have an identical learning trajectory (either based on a fixed probability of success ($p = 0.7$) or when an individual learnt (**Figure 1** of the **Main Text**)), as a function of the number of individuals sampled, and the number of replicate samples per individual. The median was always ~0; the red ($N_{replicates} = 2$) and blue ($N_{replicates} = 4$) bands are the 97.5% sampling interval for the null repeatability estimates.

Appendix 3.2 | Repeatability where there is real between-individual variation

To expand on the repeatability analyses presented in the **Main Text**, here, I present simulations in which I manipulate the distributions of N_{guess} (*i.e.*, the population values), and the learning trajectories.

I started by simulating four different distributions of N_{guess} values that differed in their mean and skewness. Specifically, using the *rnbinom* function in R, I simulated data to reflect high (size = 0.5) *versus* moderate (size = 2) right-skew, and low (mu = 15) *versus* high (mu = 30) mean. The distributional properties are presented in **Table S1**, alongside the distribution presented in the **Main Text**, and three empirical studies (Ashton et al. 2018; Harrington and Lambert 2025; McCallum and Shaw 2023).

Table S1 | Distributional properties of simulated populations used in the repeatability analyses. Note that quantiles for distributions are values of N_{guess}, and the corresponding N_{TTC} scores based on idealized TTC are shown in square brackets. Distributional parameters from three empirical studies are also shown (A et al. 18 = Ashton et al. 2018; H&L 25 = Harrington and Lambert 2025; M&S 23 = McCallum and Shaw 2023).

	mu	Size	2.5%	25%	50%	75%	97.5%
**Main Text**	**20**	**1**	**0 [10]**	**5 [12]**	**14 [32]**	**28 [33]**	**75 [74]**
Population 1	15	2	1 [10]	7 [13]	12 [19]	21 [27]	43 [48]
Population 2	15	0.5	0 [10]	1 [11]	7 [14]	20 [26]	76 [75]
Population 3	30	2	3 [11]	14 [19]	25 [30]	41 [44]	85 [85]
Population 4	30	0.5	0 [10]	3 [11]	14 [19]	40 [39]	152 [121]
A et al. 18			10	12	17	27	61
H&L 25			10	12	25	46	129
M&S 23			10	14	23	41	94

I performed repeatability simulations comparable to those described in the **Main Text**. There were six factorial conditions: $N_{individuals}$ (10, 25, 50, 100, 200), $N_{replicates}$ (2, 4), N_{learn} (0, >0), p_{max} (0.8, 1), N_{guess} (invariant, variable), and population (the above-mentioned four populations).

For N_{learn}, learning was either instant (*i.e.*, N_{learn} = 0, as per the repeatability analysis in the **Main Text**), or gradual (N_{learn} > 0, as per **Fig. 1** of the **Main Text**). For gradual learning, after N_{guess} trials at p = 0.5, p increased by 0.01 per trial, until it reached an asymptote, p_{max}.

For p_{max}, individuals mastered the task perfectly (p_{max} = 1) or made errors in 20% of trials during mastery (p_{max} = 0.8).

$N_{individuals}$, $N_{replicates}$, and N_{guess} all varied in the same way as presented in the **Main Text**.

Finally, each factorial set of parameters was replicated for each of the four populations.

As a simple example of how I constructed learning trajectories: when N_{learn} > 0 and p_{max} = 0.8 and variable N_{guess}, I sampled $N_{individuals}$ (say, 50) values of N_{guess} from one of the four populations. For one individual (with, say, N_{guess} = 8), I then sampled $N_{replicates}$ (say, 2) values of N_{guess} from 0 to 2N_{guess} (0-16). These two values of N_{guess} (say, 3 and 10) were then used to construct the learning trajectories for the two replicates (colours correspond to **Fig. 1**):

Replicate 1 | 0.5, 0.5, 0.5, 0.51, 0.52 ... 0.78, 0.79, 0.8, 0.8, 0.8, 0.8; and

Replicate 2 | 0.5, 0.5, 0.5, 0.5, 0.5, 0.5, 0.5, 0.5, 0.5, 0.5, 0.51, 0.52 ... 0.78, 0.79, 0.8, 0.8, 0.8, 0.8

Using these learning trajectories as probability vectors in the custom TTC function, I then calculated their two N_{TTC} scores. Note that I only used N_{pass} = 10 and N_{window} = 12 for these simulations. I repeated this for all individuals, partitioned variation using mixed model anova with *lme4* (Bates et al. 2015), and calculated repeatability.

I repeated this 1,000 times for all combinations producing a total of 4 x 5 x 2 x 2 x 2 x 2 x 1,000 = 320,000 estimates of repeatability.

For simplicity, I separately focused on two critical features of the design. First, I focused on the impact of sampling effort and distributional parameters on repeatability under the idealised conditions, with no biological variability (**Fig. S5**). These, therefore, set the absolute upper limit for repeatability across different population distributions. Second, I examined how biology affected repeatability for a specific set of parameters (size = 0.5, mu = 15, $N_{\text{individuals}}$ = 50, $N_{\text{replicates}}$ = 2; the lowest mean, and most skew, where repeatability is highest). I reiterate that these may not account for much of the true residual variability, but at least show how real environmental/residual variability affects repeatability (**Fig. S6**).

Despite the idealistic and biologically unrealistic parameters, repeatability estimates were highly unstable, spanning the full range of possible values (0-1) when sample sizes were low (**Fig. S5**). That is, repeatability itself is not repeatable even under idealised conditions. Increasing sample size improved the stability of repeatability estimates (albeit slowly, especially with $N_{\text{replicates}}$ = 2), but the mean repeatability stabilised around 0.85 or less (**Fig. S5**). This clearly demonstrates that increasing sample size cannot overcome the inevitably high within-individual variability that is inherent in TTC. Not only is it impossible to determine whether TTC scores themselves reflect deterministic differences *versus* stochasticity, but the magnitude of repeatability can easily be an artefact the of the method rather than a property of the individuals or traits being measured, especially if sample sizes are small ($N_{\text{individuals}}$ < 50).

I stress that these simulations (**Fig. S5**) are entirely unrealistic and idealistic. If TTC worked well, accurately estimating $N_{\text{guess}} + N_{\text{learn}} + N_{\text{pass}}$, then repeatability should consistently be 1. The only property reducing repeatability below 1 is the reliability of the measurement tool (TTC), which is unreliable owing to the stochasticity that is a property of TTC.

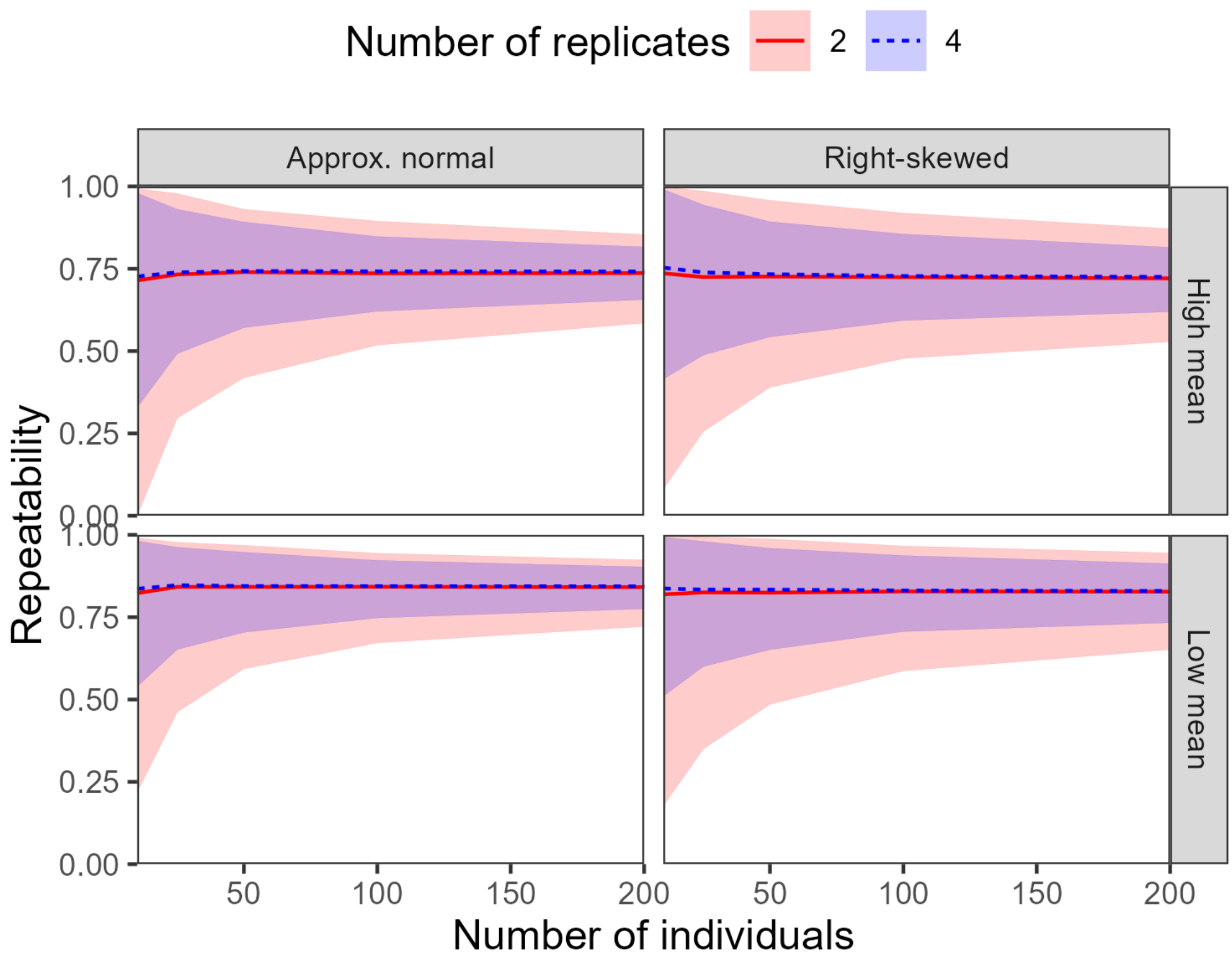

Fig. S5 | Repeatability as a function of the distributional parameters of N_{guess} (approximately normally distributed [size = 2] *versus* right-skew [size = 0.5], and low mean [15] *versus* high mean [30] for a negative binomial distribution) and sampling effort (10, 25, 50, 100, or 200 individuals, each measured twice or four times). Learning is always instant (N_{learn} = 0), perfect (*p* = 1, after task acquisition), and noise-free (identical N_{guess} across all replicates per individual). Individuals only differ in the number of guesses (N_{guess}) before reaching task acquisition. The red and blue lines [bands] represent the means [95% sampling intervals] for their respective number of replicates.

For the 50 individuals each measured twice, the mean repeatability was ~0.825 with no biological variability (**Fig. S6**, identical, instant, perfect). Adding biological noise consistently deflated repeatability estimates, and all 97.5% sampling intervals were <<1 (**Fig. S6**). Errors after task acquisition ('imperfect') had the smallest impact. With $p_{max} = 1$ ('perfect'), a task is always completed after (or within) exactly 10 trials, so +10 (or less) is consistently added. With $p_{max} = 0.8$, the probability of 10/12 passes is ~56%, and is >90% after 26 trials (**Figure S1**), so the extra variability is not extreme. Notwithstanding, the mean repeatability decreased to ~0.656, and the lower 2.5% sampling interval to ~0.310, in the range of the null distribution (**Fig. S4**). Gradual learning and variability in N_{guess} independently massively reduced repeatability, reducing the means (95% sampling intervals) to 0.514 (0.139-0.808) and 0.544 (0.201-0.840), respectively. The case with all three biological components had a very low mean of 0.361 (0.000-0.725). Ultimately, with sample sizes in the range of 50 individuals measured twice for their performance, repeatability may be extremely unreliable given any true biological noise.

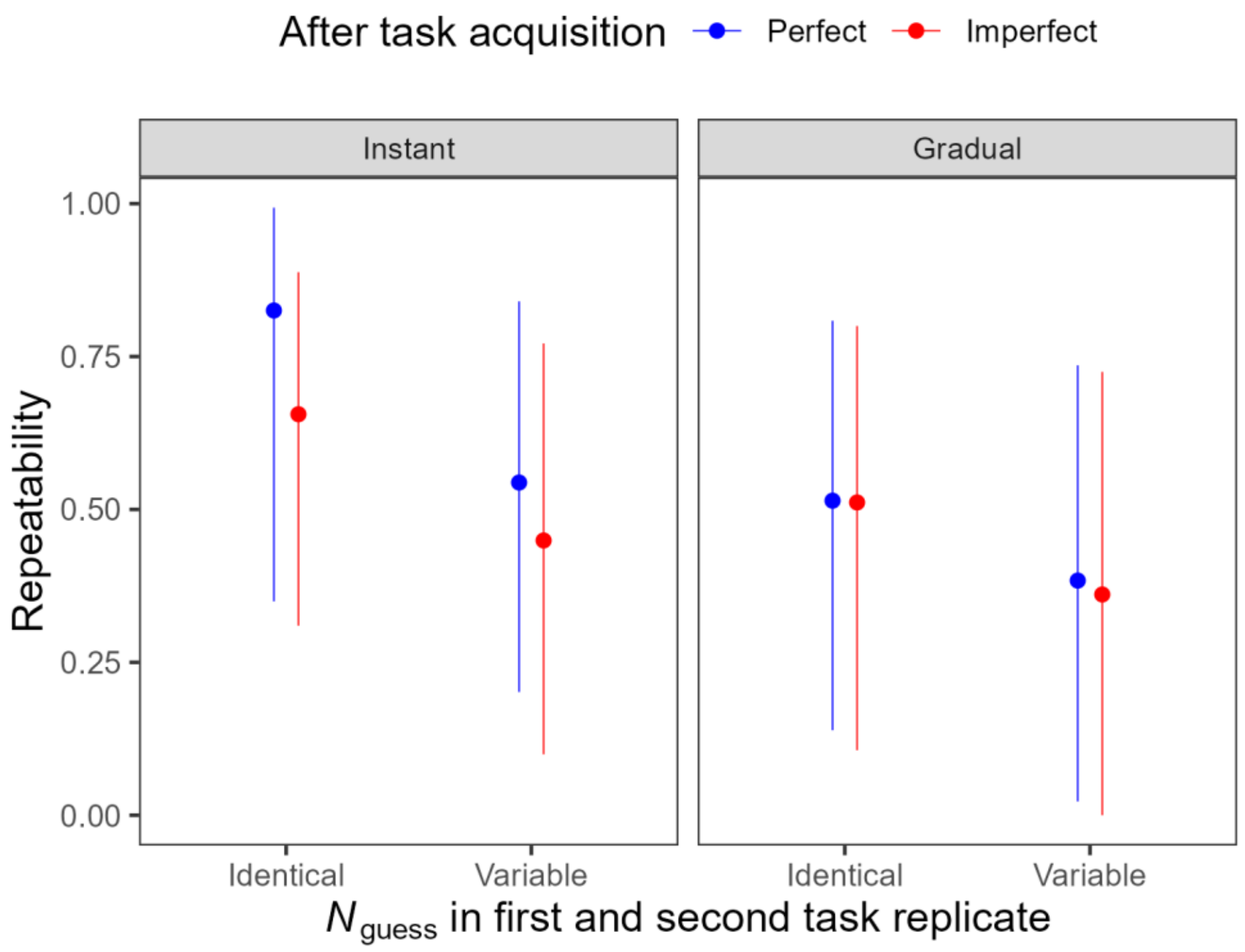

Fig. S6 | Impacts of various 'biological' effects on repeatability when 50 individuals were each measured twice for their TTC performance. Variability in the number of guesses (N_{guess}) followed a negative binomial distribution with mu = 15 and shape = 0.5. Learning was either instant (p_{min}→p_{max}) or gradual (p increased after N_{guess} trials by 0.01 per trial). For each individual, N_{guess} was either fixed or variable (see text for full details about this variability). Once learnt, individuals were either perfect ($p_{max} = 1$) or imperfect ($p_{max} = 0.8$). Points are distribution means; bars are 95% sampling intervals. Note that I chose the distributional parameters (low mean, right-skewed) of the population with the highest repeatability (**Figure S5**), such that the results reflect the 'best-case-scenario' for TTC.

Appendix 3.3 | Empirical estimates of repeatability

Generally, repeatability of TTC tasks is low (Cauchoix et al. 2018), which makes inferences from TTC difficult. Nevertheless, some empirical studies have reported high repeatability, **suggesting that TTC might be reliable**. To evaluate this possibility, I compared empirical data to those generated under idealised simulations.

Using the 'population' described in the **Main Text**, I sampled 100 individuals (*i.e.*, 100 independent values of N_{guess}). For each individual, I constructed their learning trajectory under idealised conditions (p = 0.5 for N_{guess} trials; p = 1 for 10 trials thereafter; see **Main Text**), and then simulated two N_{TTC} scores using the TTC function.

For these 100 simulated individuals, their two replicate scores are represented by the grey crosses in **Fig. S7**. Despite using the identical learning trajectory for both replicates, most crosses deviate from the dashed 1:1 line. This predominately arises from stochastic variation in the few trials immediately preceding task acquisition. For instance, consider an individual with N_{guess} = 25 (*i.e.*, an individual that has exactly 25 guesses each time they attempt a task). In their first replicate, they fail (X) trials 23-25 (XXX), before acquiring the task and completing a sequence of 10 passes (O), producing a score 35. In their second replicate, their last few pre-acquisition trials included four passes (XOOXOXO); thus, only six more passes were required for 10/12, resulting in a score of 31. **Stochasticity in the pre-acquisition trials leads to the expectation that, *at best*, repeated measurements for *most* individuals will differ by several trials**. Occasionally, individuals may also succeed within the guessing phase ('false positives'), generating larger score discrepancies (*e.g.*, one individual scores ~120 and then ~12; **Fig. S7**). These two phenomena – that together explain both the minor jitter and the extreme scatter of the grey points around the 1:1 dashed line – are inherent in two-choice TTC. The grey points are a representation of the minimum scatter that is theoretically possible under a two-choice, 10/12 TTC design.

These simulated data are plotted alongside empirical data from two studies that report high repeatability. Strikingly, the N_{TTC} scores from Ashton et al. (2018) fall close to the 1:1 line, with dramatically less scatter than the theoretical lower bound (blue dots, **Fig. S7**). This level of consistency is incompatible with both stochastic phenomena described above, and, therefore, implies the presence of a mechanism that suppresses within-individual variation. Although the nature of such a mechanism remains unclear, any process that reduces within-individual variation to this extent necessarily **undermines the objective of TTC and the inferences that depend on it** (see the **Main Text**). Consequently, rather than providing evidence that TTC is reliable, these empirical results may instead reveal another way in which TTC is remarkably unreliable.

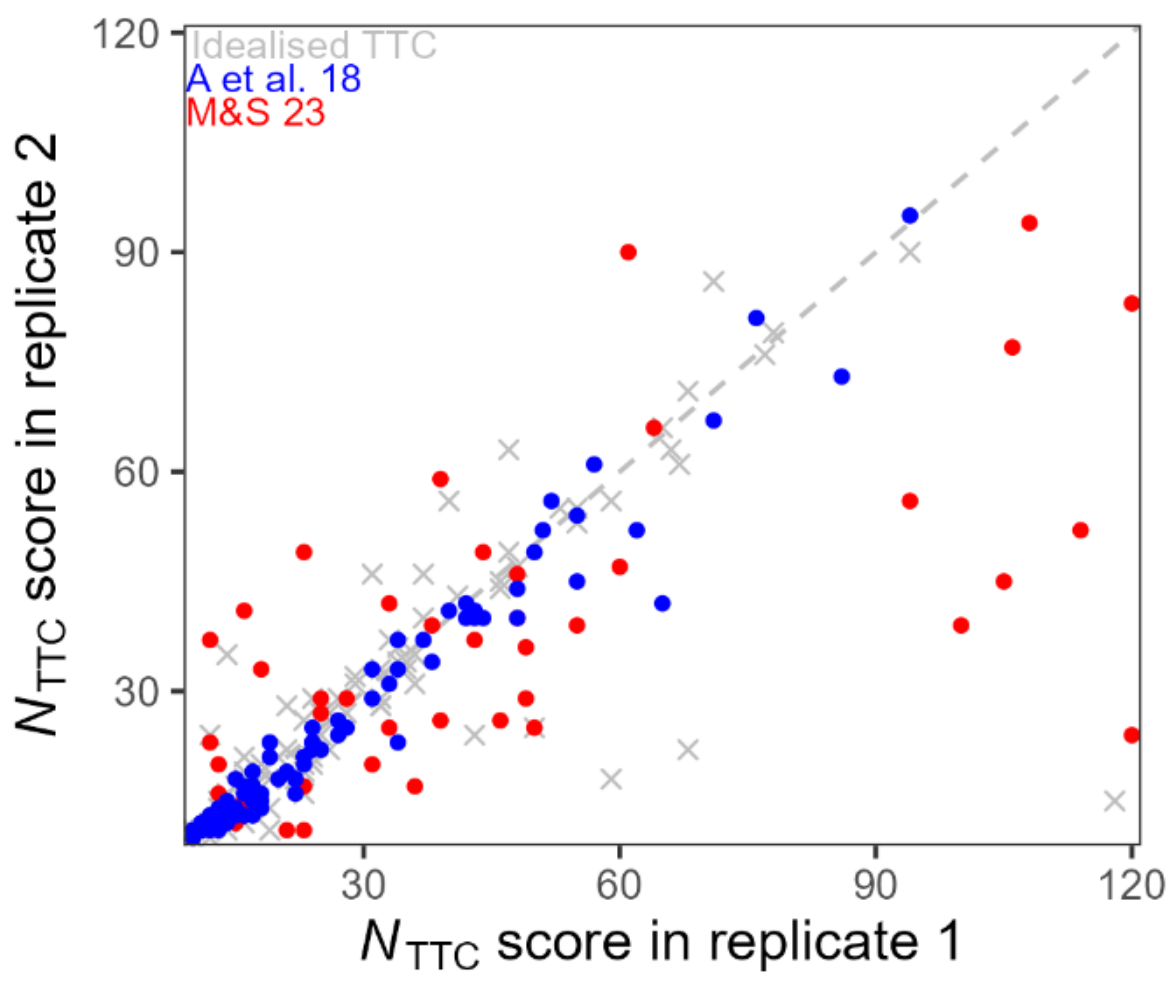

Fig. S7 | Number of trials to reach criterion (N_{TTC}) in two replicates for empirical (red: McCallum and Shaw (2023); blue: Ashton et al. (2018)) and simulated data (grey crosses). Each empirical study measured two replicates per individual for each of two tasks (associative learning and reversal learning). Simulated data represent two replicates for 100 individuals under the idealised scenario described in the **Main Text**. Each individual guessed (p = 0.5) for their specific, fixed number of trials (identical in both replicates), before acquiring the task and being perfect thereafter (p = 1). Across all scenarios, N_{pass} = 10 and N_{window} = 12. The dashed line is 1:1. The 100 simulated data points represent a repeatability of ~0.8, which is the **mean** repeatability under idealistic conditions. The near-perfect repeatability in one empirical study sets an unrealistic expectation for TTC studies and falsely suggests that TTC is almost perfectly reliable.

Appendix 4 | Reducing the false positive risk

In the **Main Text**, I showed that the 10/12 task parameters could result in higher repeatability than the 5/5 task parameters; stochasticity is reduced because the per-window probability of success is lower, which reduces the false positive risk. Consequently, tasks are less likely to be completed by chance, and, thus, individuals are more likely to reach task acquisition (true positive), and their scores become more consistent. A larger contribution by the deterministic reaching of the task criterion ensures that scores are more repeatable.

Here, I demonstrate how minimising the false positive risk can make scores more accurate and precise (*i.e.*, directly look at scores, not repeatability). I performed the same analyses as **Appendix 2.2**, but altered the **minimum probability of success to p = 1/3, mimicking a task with three options instead of two**.

N_{TTC} scores were dramatically more accurate and precise with a three-option task. Although mean and median scores failed to track the red line when learning was not instant (not perfectly accurate), precision across the range of N_{guess} values at least ensures that the method could theoretically separate true between-individual differences from stochasticity. However, while minimising stochastic reaching of the task criterion could theoretically improve TTC, the risk of false negatives may become problematic (among other things; see **Main Text**). In a realistic, empirical study, where individuals make errors, setting strict criteria and inflating false negatives could be more problematic than less stringent criteria and an inflation of false positives.

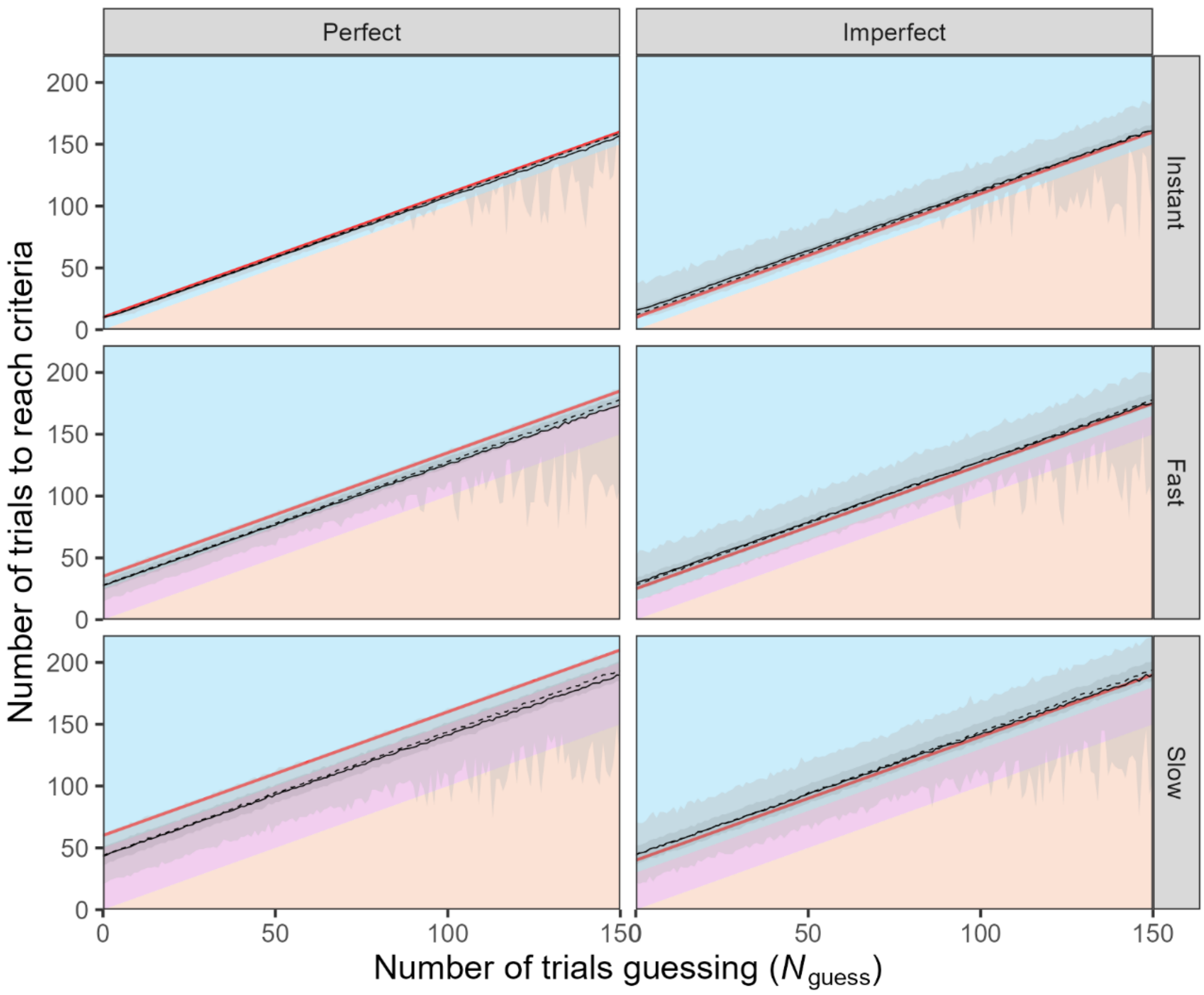

Fig. S8 | Number of trials to reach criteria as a function learning speed (instant, fast, or slow) and accuracy (perfect [p_{max} = 1] or imperfect [p_{max} = 0.8] once 'learnt') parameters. Across 1,000 simulations per parameter set and value of N_{guess} (number of guesses before learning): black line is the mean score; dashed line is the median score; dark grey band is the interquartile range (25-75%); light grey band is the 95% sampling intervals (2.5-97.5%). Background colours represent the three phases (guessing = orange, learning = pink, mastery = light blue) in the associated trial number (see **Figure 1** in the Main Text). The red line represents N_{guess} + N_{learn} + N_{pass}, which is possibly the 'ideal score' captured by TTC (see text, above). Accuracy of the method is represented by how closely the median and mean track the red line, while precision is represented by how tightly the 50% and 95% quantiles straddle median/mean. Unlike **Figure S2**, where there are two options (50% probability of success in each trial), here, there are three options, and a ~33% probability of success in each trial.

Appendix 5 | Heritability power analysis

Appendix 5.1 | Parent-offspring regression

I simulated N_{TTC} data, and performed parent-offspring regression analyses, to determine how TTC might impact the power to detect heritability (h^2).

I started by creating a population where a trait had phenotypic variance of $V_P = 400$ and a mean of $\mu = 15$. These values are somewhat arbitrary, but ultimately produce a skewed distribution of N_{TTC} scores that is common in TTC research. Additive genetic variance was $V_A = h^2V_P$ and residual variance $V_E = (1 - h^2)V_P$. Using the *rnorm* function, I sampled sire (A_{sire}) and dam (A_{dam}) breeding values, and sire (E_{sire}) and dam (E_{dam}) environmental deviations, from normal distributions with $N(0, V_A)$ and $N(0, V_E)$, respectively. Sire and dam latent phenotypes were then calculated as the sum of the breeding value, the environmental deviation, and the trait mean. Latent phenotypes were then rounded and truncated at 0; these represent the N_{guess} values.

Offspring breeding values (A_{off}) were sampled by first calculating the midparent breeding value ($A_{mid} = 0.5*A_{sire}*A_{dam}$), and then sampling a single value from a normal distribution with $N(A_{mid}, \sqrt{(0.5*V_A)})$. Rather than simply assign $A_{off} = A_{mid}$, this step incorporates Mendelian sampling variance into the offspring breeding value. As above, I then rounded and truncated phenotypes to generate N_{guess} values.

For all individuals, I then calculated their observed N_{TTC} scores using the custom TTC function presented in the **Main Text**, with $N_{pass} = 10$, $N_{window} = 12$. Like the **Main Text**, N_{TTC} scores were calculated in different conditions: idealised (N_{guess} was the exact, sampled value, and $p_{max} = 1$) and empirical-like (N_{guess} was determined by sampling from $N(N_{guess}, (N_{guess}/1.96)^2)$, and $p_{max} = 0.85$).

Hence, all individuals have four phenotypes: their latent quality (standard, Gaussian data, with broad applicability for statistical power in parent-offspring heritability analyses beyond TTC studies), their rounded and truncated N_{guess} (thus allowing us to separate the effects of a skewed distribution from the stochastic variability of TTC itself), and their idealised and empirical-like N_{TTC} scores.

Using either one parent (the sire) or the midparent value, I then performed a regression analysis (offspring on parent values) to estimate the slope of the relationship, doubled for the sire-only analysis, which provided an estimate of h^2. While I did not simulate any maternal effects, these are well-known to seriously inflate dam-offspring resemblance and confound genetic and environmental sources of variation ('nature' *versus* 'nurture'). I explicitly analysed sire-offspring regression because dam-offspring regression should generally be considered 'pseudo-heritability'.

I performed one regression analysis per 'trait'; four in total for each simulated data set. From each model, I also recorded the *p*-value.

I repeated this analysis 1,000 times for each trait and the following fully factorial parameters: sire *versus* midparent, $h^2 = 0.1$, 0.2, 0.4, 0.8, and $N_{relationships} = 10$, 25, 50, 100, 200, 500, generating a total of 4 x 2 x 4 x 6 x 1,000 = 192,000 *p*-values and estimates of h^2. For each parameter set, I calculated the interquartile ranges and 95% sampling intervals of the sampling distribution of h^2. Additionally, to evaluate statistical power, I recorded the proportion of experiments where $p < 0.1$ and $h^2 > 0$. Note that I use $p < 0.1$ to reflect one-tailed tests, since V_A and h^2 have lower bounds of 0.

Latent phenotype analyses (maroon in **Fig. S9** and **S10**) provide general insight into sampling distributions and statistical power for quantitative traits. Although countless analytical and simulation-based power analyses (see the **Main Text**) exist for heritability estimation, I present my results, here, to provide a baseline against which the other 'trait' types can be compared. Notably, h^2 estimates from midparent-offspring and sire-offspring analyses were unbiased, with median estimates all approximately on the dashed lines (**Fig. S9**). Also, sire-offspring analyses were substantially weaker than midparent-offspring analyses, and very small sample sizes ($N \leq 25$ for midparent-offspring, $N \leq 50$ for sire-offspring) result in extremely wide 95% sampling intervals (**Fig. S9**), ensuring h^2 estimates derived from studies of this scale are, not only powerless (**Fig. S10**), but are practically uninformative.

Estimates of h^2 based on N_{guess} phenotypes (censored, rounded) were only marginally downwardly biased. The downward bias was more substantial for TTC phenotypes: estimates were deflated by ~25% under idealised conditions, and >50% when 'biological' variability was included, relative to the latent phenotype analyses. Statistical power is substantially weaker with TTC. N = 500 midparent-offspring relationships only just had ~80% power to detect h^2 = 0.4. However, to reach 80% power to detect h^2 = 0.2, N >>> 500 is required (thousands from sire-offspring analyses). While estimates from well-replicated experiments ($N \geq 200$) are relatively precise (minimal sampling variance of h^2), albeit downwardly biased, for latent phenotypes, rounded and truncated N_{guess} scores, and idealised N_{TTC} scores, they remain highly variable when there is variability in N_{guess}, particularly for sire-offspring analysis. Unless there are at least thousands of parent-offspring relationships, TTC may not be able to meaningfully estimate heritability.

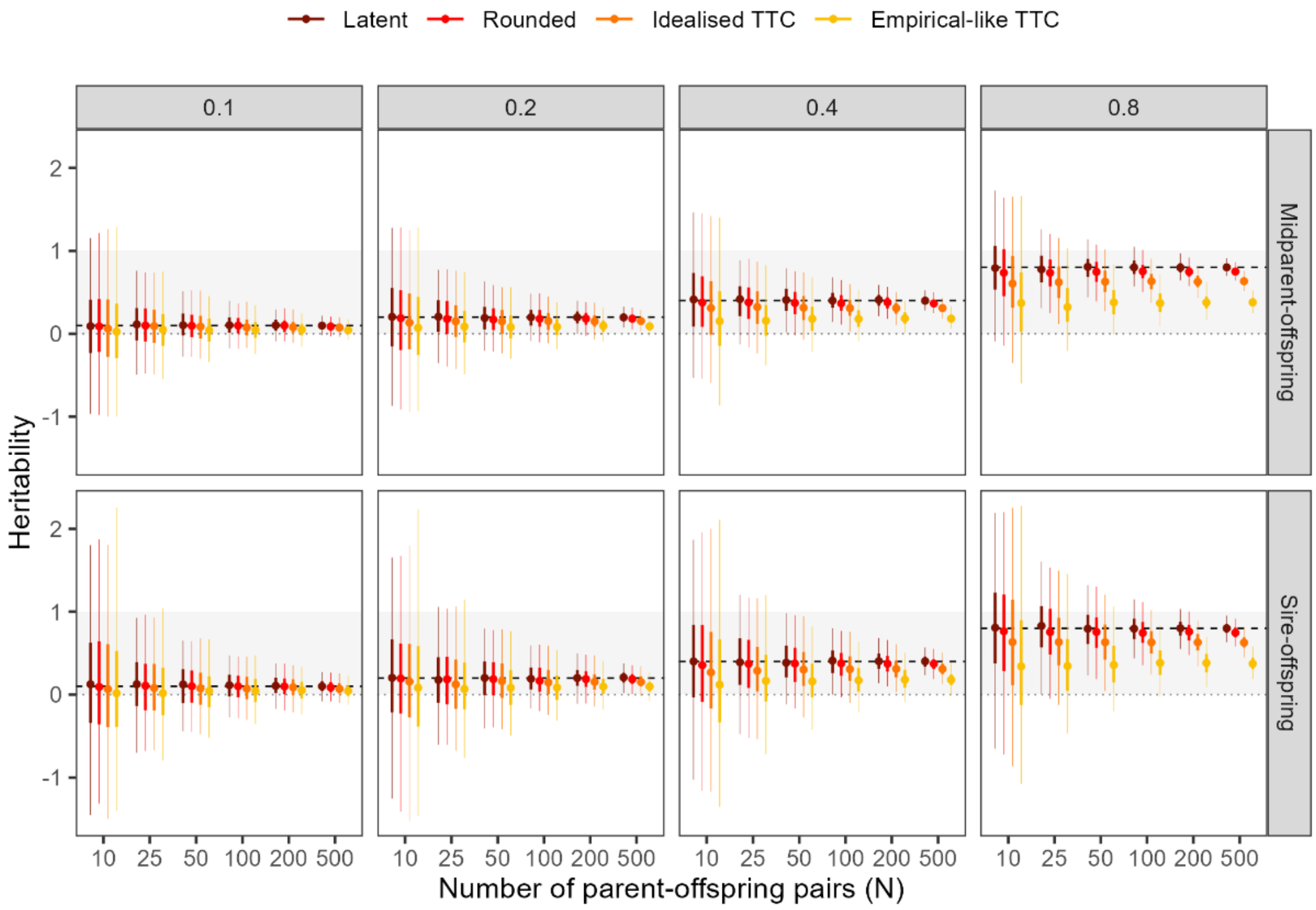

Fig. S9 | Distribution of heritability (h^2) estimates from parent-offspring regression. Points are the median estimates, thick bands are interquartile ranges, and thin bands are 95% sampling intervals. Black dashed lines are the simulated, true heritability; grey dotted lines are at 0; light grey box represents the range of possible h^2 values (0-1).

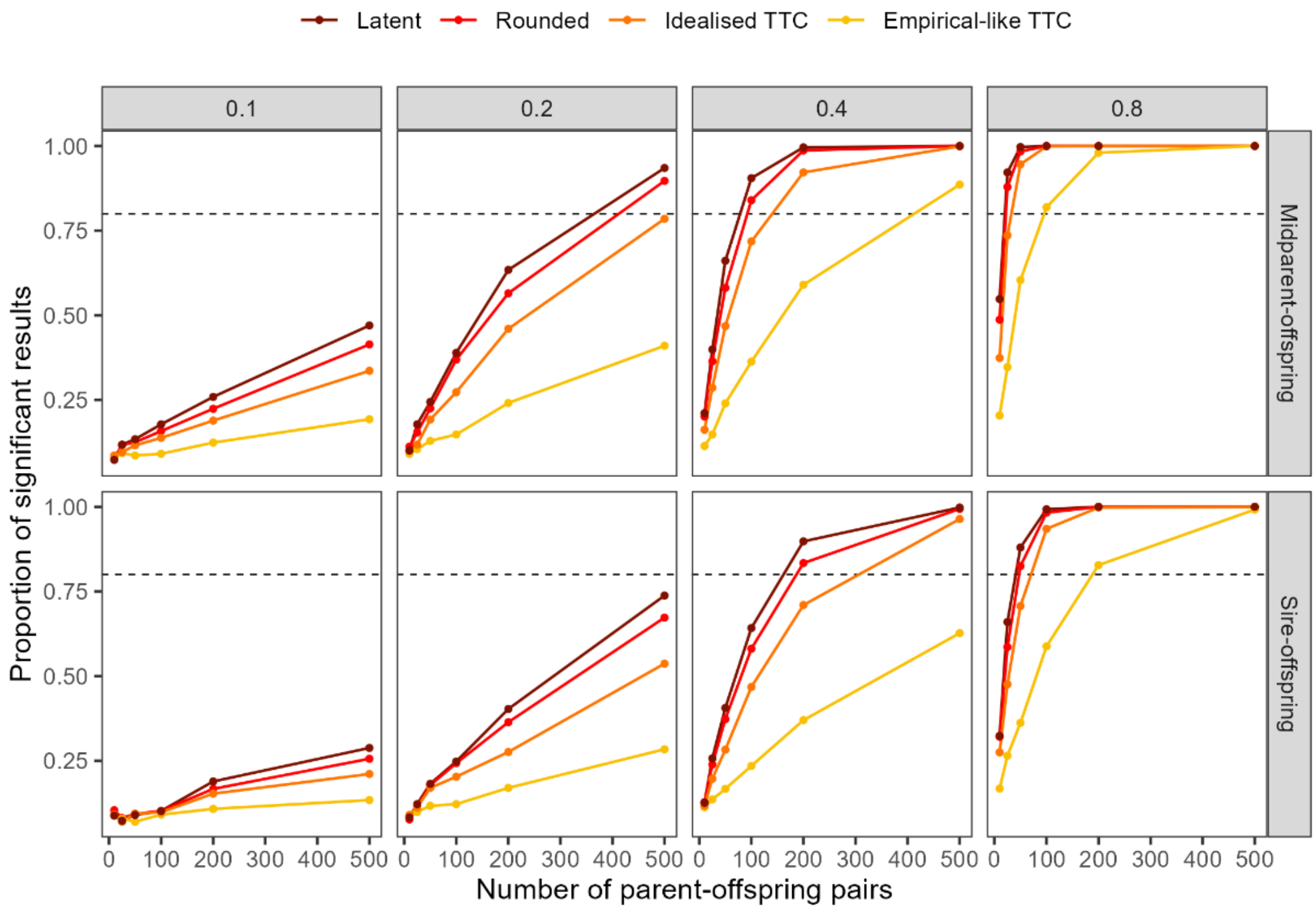

Fig. S10 | Power to detect heritability. Dashed line represents 80% power, generally considered to be a well-powered experiment. Points below the black dashed line represent underpowered experiments for the given sample size (N) to detect the associated heritability.

Appendix 5.2 | Full-sibling half-sibling, sire-dam, mixed model analysis

Another common method for estimating heritability is variance partitioning through full-sib half-sib breeding designs. I simulated breeding designs where 10, 25, 50, 100, 200 sires (N_{sire} = 10, 25, 50, 100, or 200) were each mated to three dams, and each dam produced either five or 10 offspring ($N_{offspring}$ = 5 or 10). I followed the exact procedures outlined above to calculate sire, dam, and offspring breeding values, environmental deviations, and latent phenotypes. Once the offspring latent phenotypes were calculated, I then calculated their N_{guess} values (rounding and truncating), and simulated their idealised and empirical-like N_{TTC} scores. I only used offspring phenotypes in the analysis.

I partitioned variation with a random effects model, implemented using the *lme4* package (Bates et al. 2015) in R. Sire and dam (nested within sire) were random effects, with offspring phenotypes the response variable. Additive genetic variance (V_A) is four times the among-sire variance (V_S), and heritability (h^2) is the proportion of variance attributable to V_A: $h^2 = \frac{4V_S}{V_S+V_D+V_E}$. To test statistical significance, I performed a likelihood ratio test, where twice the difference in log-likelihoods between nested models that included or excluded sire as a random effect are tested against the χ^2 distribution with one degree of freedom. I recorded the proportion of values where $p < 0.1$, again reflecting the one-tailed nature of variance estimation. This produced a total of 4 x 4 x 5 x 2 x 1,000 = 160,000 p-values and estimates of h^2.

Again, in these simple simulations, I could have equally used among-dam variance, but this is bad practice. Typically, among-dam variance usually includes: maternal effects, shared environmental effects, and non-additive genetic effects (dominance), and is thus inflated relative to among-sire variance.

The results are presented in the same way as in **Appendix 5.1**. Ultimately, these results strongly suggest that estimating the heritability of cognitive traits with TTC not be attempted under most (probably all) scenarios.

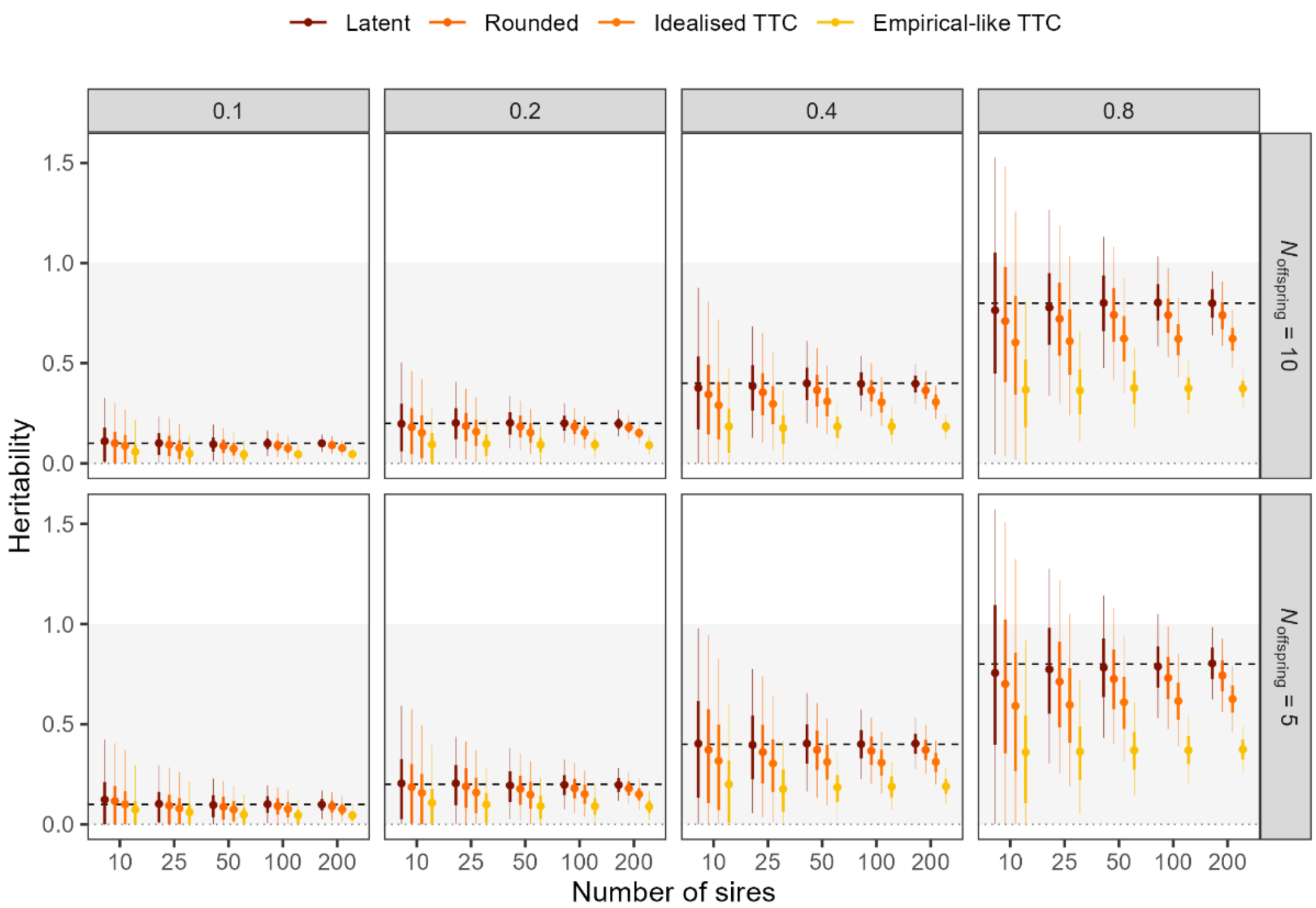

Fig. S11 | Distribution of heritability (h^2) estimates from full-sibling half-sibling mixed model analysis. Points are the median estimates, thick bands are interquartile ranges, and thin bands are 95% sampling intervals. Black dashed lines are the simulated, true heritability; grey dotted lines are at 0; light grey box represents the range of possible h^2 values (0-1).

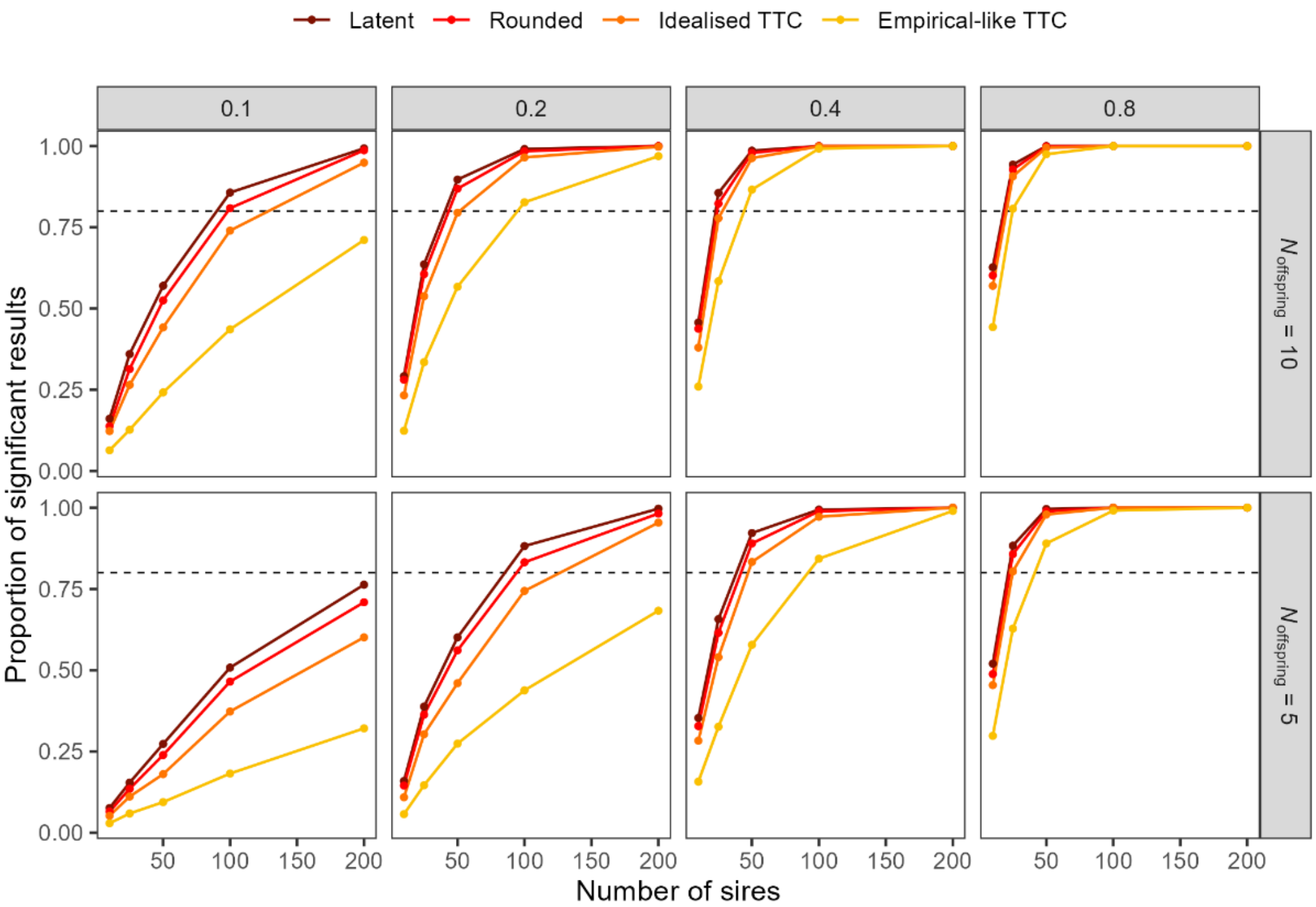

Fig. S12 | Power to detect heritability. Dashed line represents 80% power, generally considered to be a well-powered experiment. Points below the black dashed line represent underpowered experiments for the given sample size (*N*) to detect the associated heritability.

Appendix 6 | The criterion undermines the slope analysis

In their recent paper, Harrington and Lambert (2025) used trial-by-trial data to overcome some of the fundamental pitfalls of TTC. Birds (caracara) were tested using the 10/12 TTC method. A core part of the data analysis involved calculating the slope of the logistic regression relationship between trial number [independent variable] and trial success (pass/fail; 1/0) [dependent variable] for each individual. Intuitively, if the probability of passing increases (*i.e.*, if an individual is learning), so too should the proportion of passes through time, and the slope of the logistic regression relationship between trial success (0/1) and trial number should be positive.

As a necessary step to confirm whether slopes exceeded what could be expected by random chance (*i.e.*, to confirm that a task has been truly learnt, and not completed by luck), they then generated a null distribution of slopes by simulating random agents against which the empirical slopes were compared. Specifically:

"To evaluate whether observed slopes exceeded chance, we simulated individual-specific null distributions. For each bird, we simulated 1000 random agents ($p = 0.5$) over the same number of trials, computed logistic slopes and calculated p-values as the proportion of null slopes ≥ observed. We considered slopes falling within the top 5% of the individual's null distribution ($p < 0.05$) significant."

Ten of 32 birds had statistically significant positive slopes for associative learning.

Unfortunately, I believe the logic is slightly flawed. The null distribution produced by this method will be symmetrically distributed about zero (**Fig. S13**); just as many slopes will be negative as they would be positive. **Crucially, in TTC, regardless of whether the criterion is met by learning (true positive) or chance (false positive), a task finishes after a run of successes (in this case, 10 out of 12)**. Therefore, by definition, the average probability necessarily reaches its zenith within the last moving window of trials. Consequently, the slope of the logistic regression curve will *almost always be positive*. If the objective is to distinguish slopes that result from learning *versus* chance, then the null distribution described by Harrington and Lambert (2025) is incorrect; it does not account for the condition of TTC that the last 12 trials **must have 10 successes** (except in cases where the task ends before the twelfth trial), which forces positive slopes and ensures that their slope test is anti-conservative. It is equivalent to comparing the mean height of children to the mean height of those tall enough to ride the rollercoaster.

To illustrate this, I simulated 'random agents' of known lengths (between 12 and 30; N_{agent}) that were either entirely random, or with the condition that the final 12 trials had to contain 10 passes. The former simply involved sampling N_{agent} values of 0 or 1 using the *rbinom* function, with $p = 0.5$ (*i.e.*, exactly in line with Harrington and Lambert (2025)). For the latter, I ran the custom TTC function with $p = 0.5$ (*i.e.*, a fixed vector where 0/1 outcomes were completely random, and the criterion were met by 'luck'), but instead of storing the N_{TTC} score, stored the trial-by-trial outcomes. I then truncated the vector of trial-by-trial outcomes to include only the last N_{agent} values (note that if the vector was shorter than N_{agent}, I simply re-ran that simulation). For each set of scores (random [unconditional] or TTC [conditional]), I then computed the logistic regression slopes using binomial generalised linear regression with *glm* in R (R Core Team 2025). For each value of N_{agent}, I performed 10,000 simulations, producing a total of 19 x 2 x 10,000 = 380,000 logistic regression slopes.

Additionally, using the trial-by-trial data for each of 32 birds from Harrington and Lambert (2025), I calculated their observed logistic regression slopes. I then used the corresponding number of trials (N_{TTC}) and above-mentioned simulation approaches to compare the observed slopes to the null distributions generated from the conditional and unconditional methods. A significant deviation from the null was when the observed slope was in the top 5% of the null.

Unsurprisingly, the null distribution, conditioned on the TTC 'endpoint', was systematically biased to be positive, with the upper 97.5% sampling interval consistently much higher than the unconditioned method (**Fig. S13**). Consequently, of the 10 birds identified to have significantly positive slopes (the same 10 birds identified by Harrington and Lambert (2025)), only one was significantly positive based on the conditional method (*i.e.*, 3.125% of significant slopes; **Table S2**); exactly as expected given that the false positive error rate was set at 5%.

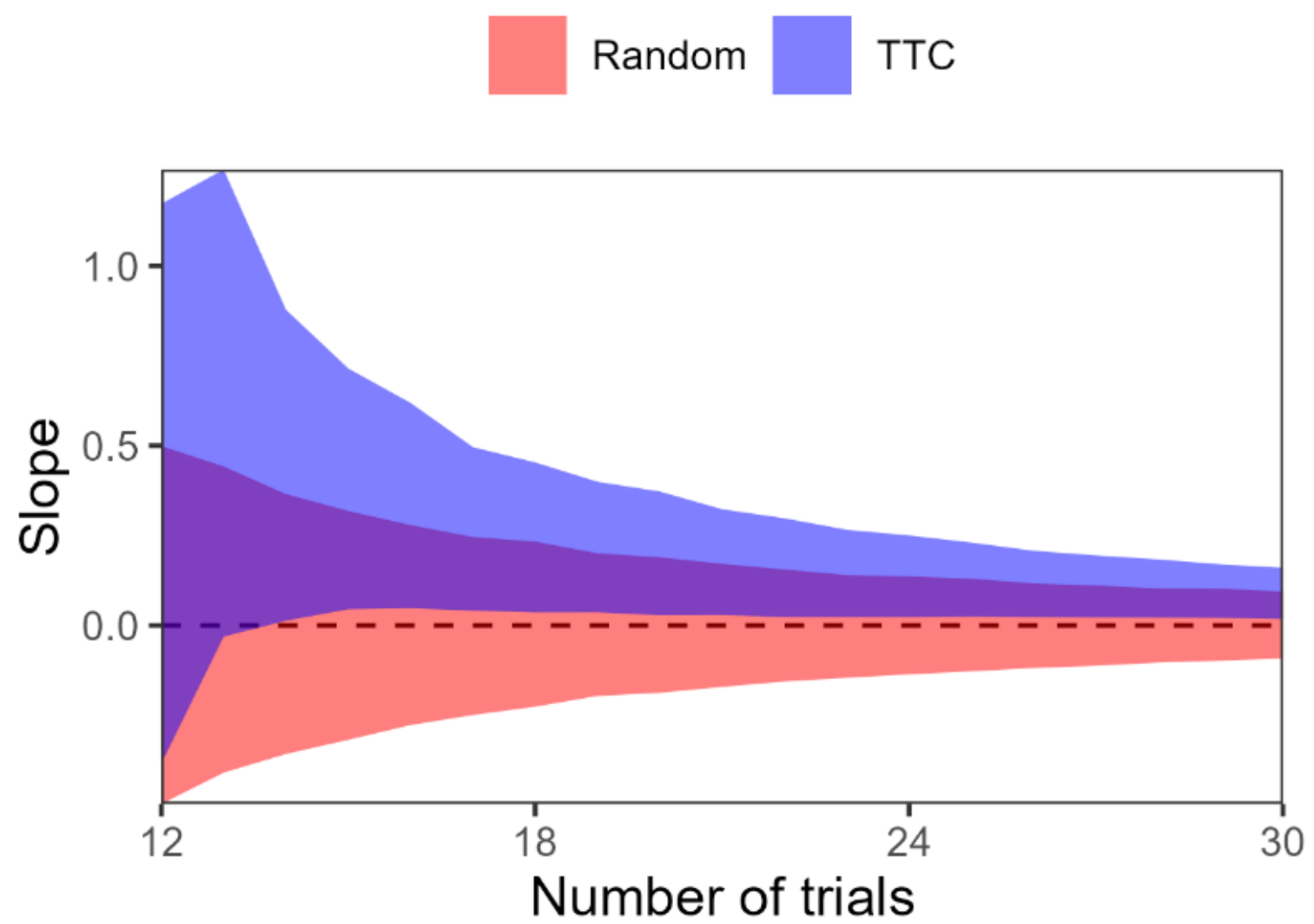

Fig. S13 | Null distributions for logistic regression slopes generated from 'random agents' of varying lengths (number of trials). For each number of trials (N_{agent}), I generated either an unconditional sequence of Bernoulli outcomes (i.e., 0 or 1) that represent the null distribution ('Random') used by Harrington and Lambert (2025), or a conditional random sequence, where the final 12 elements contained exactly 10 values of one ('TTC'). Logistic regression slopes were computed for each sequence type, and the simulation was repeated 10,000 times for each N_{agent}. The shaded areas represent the 95% sampling intervals for the corresponding distributions. While the unconditioned null is symmetrically distributed about zero, the TTC condition shows systematically positive slopes that are much higher for any given number of trials. Crosses are slopes observed by Harrington and Lambert (2025).

Table S2 | Observed logistic regression slopes and their corresponding p-values under the unconditional (p_{unco}) and conditional (p_{con}) null models (only birds with $p_{unco} < 0.05$ are shown).

Bird ID	N_{TTC}	Slope	p_{unco}	p_{con}
H60	129	0.010	**0.017**	0.075
K33	66	0.033	**0.007**	0.077
R34	20	0.212	**0.016**	0.256
S33	37	0.057	**0.046**	0.422
S34	21	0.140	**0.047**	0.534
U13	43	0.045	**0.037**	0.400
U34	48	0.042	**0.034**	0.283
V11	22	0.172	**0.021**	0.258
V34	12	0.712	**0.009**	**0.042**
Y14	42	0.078	**0.003**	0.054

Ultimately, TTC ends after a high proportion of successes, whether learning happens or not. Any null distribution must account for this, or risks over-interpreting results (*e.g.*, may conclude that there are true differences between birds with significantly positive slopes *versus* the other 22 birds). I am not suggesting that the null that I present is a good idea; indeed, **any slope analysis may be flawed when the data are conditioned on a task criterion**. Standard binomial sampling, where there is fixed number of trials per individual, coupled with the unconditional null proposed by Harrington and Lambert (2025), would allow hypothesis testing of the observed slope and intercept. This might be particularly powerful if combined with a design that also reduces the per-trial probability of success (*e.g.*, three options; **Fig. S8**), or for reversal learning tasks where failure in early trials is expected (Harrington and Lambert 2025).

Finally, it is worth explicitly stating that the inference is not that birds are not learning; they clearly are, or else the median N_{TTC} score would be >100 (**Fig. S2**), not ~25. The issue is in making inferences about ***inter-individual*** differences in cognitive abilities. A single learning trajectory can generate a broad distribution of N_{TTC} scores purely through the stochasticity of Bernoulli sampling. The observed distribution of N_{TTC} scores for the caracaras could easily be reproduced by a single learning trajectory (e.g., **Fig. S2**). Harrington and Lambert (2025) recognise this, motivating their shift away from TTC and towards analysing the logistic slope. Unfortunately, their new analysis does not account for the condition of TTC that essentially forces a positive slope, and so cannot resolve the fundamental problem (TTC obscures inferences about true between-individual differences). The criterion undermines the slope analysis.

Appendix 7 | Custom TTC function

Below is the custom TTC function used to calculate N_{TTC} scores. Underneath is an example usage demonstrating that more complex designs (five choices [random success is 0.2], 16/20 criterion) avoid the false positives problem, but still produce a wide range of scores that will inevitably swamp between-individual differences.

```
### Function to simulate trials-to-criterion (TTC) task
## Inputs:
# Npass: the number of passes required within a window size of Nwindow (default is 10)
# Nwindow: moving window size, within which Npass passes must be achieved to reach the task criterion (default is 12)
# p: vector that defines the learning trajectory; the ith p is the probability of success in the ith trial (default is 10000 at p = 0.5)
TTC <- function(Npass = 10,
          Nwindow = 12,
          p = rep(0.5, 10000)){
 max_iter <- length(p)
 window <- integer(0)
 trials <- 0
 repeat{
  trials <- trials + 1
  if(trials > max_iter) break
  window <- c(window, rbinom(1, 1, p[trials]))
  if(length(window) > Nwindow) window <- tail(window, Nwindow)
  if(sum(window) >= Npass) break
 }
 return(trials)
}

# Scores for a 16/20 task where the probability of passing by chance is 0.2, learning is gradual, and task acquisition produces 80% success
set.seed(1)
LearningTrajectory <- c(rep(0.2, 15), seq(0.24, 0.8, 0.04), rep(0.8, 10000))
NTTC <- replicate(1000, TTC(16, 20, LearningTrajectory))
hist(NTTC, breaks = 100)
range(NTTC)
```

References

Ashton BJ, Ridley AR, Edwards EK, & Thornton A (2018) Cognitive performance is linked to group size and affects fitness in Australian magpies. Nature 554(7692):364-367.

Bates D, Mächler M, Bolker B, & Walker S (2015) Fitting linear mixed-effects models using lme4. Journal of Statistical Software 67:1-48.

Borsboom D (2008) Latent variable theory. Measurement 6:25-53.

Cauchoix M, Chow P, Van Horik J, Atance C, Barbeau E, Barragan-Jason G, Bize P, Boussard A, Buechel SD, & Cabirol A (2018) The repeatability of cognitive performance: a meta-analysis. Philosophical Transactions of the Royal Society B: Biological Sciences 373(1756):20170281.

Dohm M (2002) Repeatability estimates do not always set an upper limit to heritability. Functional Ecology 16(2):273-280.

ESPNcricinfo (2026). Mitchell Starc batting and bowling statistics. https://www.cricinfo.com/cricketers/mitchell-starc-311592. Accessed August 5, 2025.

Harrington KJ, & Lambert ML (2025) Beyond criterion: cognitive flexibility in wild striated caracaras. Biology Letters 21(11):20250495.

McCallum E, & Shaw RC (2023) Repeatability and heritability of inhibitory control performance in wild toutouwai (*Petroica longipes*). Royal Society Open Science 10(11):231476.

R Core Team (2025) 'R: A Language and Environment for Statistical Computing'. Vienna, Austria: R Foundation for Statistical Computing. Available at: https://www.R-project.org/.
